# Effect of gga-miR-155 on cell proliferation, apoptosis and invasion of Marek’s disease virus (MDV) transformed cell line MSB1 by targeting RORA

**DOI:** 10.1186/s12917-020-2239-4

**Published:** 2020-01-28

**Authors:** Ke Ding, Zu-Hua Yu, Chuan Yu, Yan-Yan Jia, Lei He, Cheng-Shui Liao, Jing Li, Chun-Jie Zhang, Yin-Ju Li, Ting-Cai Wu, Xiang-Chao Cheng, Zi-Yu Zhou, Zu-Ling Yu

**Affiliations:** Key Laboratory of Animal Disease and Public Health, Henan University of Science and Technology, Luoyang, 471003 People’s Republic of China

**Keywords:** Gga-miR-155, Marek’s disease, MSB1, Lymphocyte line, Proliferation, Apoptosis, RORA

## Abstract

**Background:**

Marek’s disease (MD) is caused by the oncogenic Marek’s disease virus (MDV), and is a highly contagious avian infection with a complex underlying pathology that involves lymphoproliferative neoplasm formation. MicroRNAs (miRNAs) act as oncogenes or tumor suppressors in most cancers. The gga-miR-155 is downregulated in the MDV-infected chicken tissues or lymphocyte lines, although its exact role in tumorigenesis remains unclear. The aim of this study was to analyze the effects of gga-miR-155 on the proliferation, apoptosis and invasiveness of an MDV-transformed lymphocyte line MSB1 and elucidate the underlying mechanisms.

**Results:**

The expression level of gga-miR-155 was manipulated in MSB1 cells using specific mimics and inhibitors. While overexpression of gga-miR-155 increased proliferation, decreased the proportion of G1 phase cells relative to that in S and G2 phases, reduced apoptosis rates and increased invasiveness. However, its downregulation had the opposite effects. Furthermore, gga-miR-155 directly targeted the RORA gene and downregulated its expression in the MSB1 cells.

**Conclusion:**

The gga-miR-155 promotes the proliferation and invasiveness of the MDV-transformed lymphocyte line MSB1 and inhibits apoptosis by targeting the RORA gene.

## Background

Marek’s disease virus (MDV) is an oncogenic herpesvirus, reclassified as the Gallid alphaherpesvirus 2 (GaHV2) [1]. It is the causative agent of Marek’s disease (MD), which is characterized by complex clinical syndromes, including immune suppression, paralysis, neurological signs and lesions, and the rapid formation of CD4^+^ T-cell lymphomas [2–4], and is responsible for considerable losses to the poultry industry worldwide. Although a vaccine is available against MD, eruptions are common even in immunized chicken flocks, likely due to intensive farming, incomplete immunization and increasing virulence [5–8]. Therefore, it is essential to determine the molecular mechanisms underlying MDV-induced oncogenesis.

MicroRNAs (miRNAs) are endogenous small non-coding RNA that degrade mRNAs or inhibit translation by binding to their 3′-untranslated regions (3′-UTR), and therefore regulate multiple cellular processes, such as proliferation, cell cycle, apoptosis, migration and metabolism [9–13]. Recent studies have identified several host and viral miRNAs that potentially regulate MDV-induced tumorigenesis [14–17]. MiRNA-155 is a conserved multifunctional cellular miRNA that regulates the proliferation, migration and invasive growth of tumor cells, and is therefore closely associated with tumor initiation and progression [18–21], Studies show that the MD-encoded miR-M4-5p is the viral ortholog of cellular miR-155, and shares common targets with miR-155 and is involved in MD lymphomagenesis [16, 17, 22, 23]. Zhao et al. have implicated the critical role of MDV-miR-M4 in the induction of tumors, demonstrated the similarities function of both orthologs [24]. However, recent studies on MDV-transformed cell lines suggest that continued expression of miR-M4 is not essential to maintain the transformed phenotype [25, 26].

MDV-miR-M4 is highly expressed in virus-infected CEFs, MDV-induced tumor tissues, lymphoblastoid cell lines and serum exosomes [4, 27, 28], and promotes MDV tumorigenesis. In contrast, miR-155, gga-miR-181a and gga-miR-26a are downregulated in MDV-transformed T lymphocyte lines, MDV-induced tumors and MDV-infected peripheral blood lymphocytes [29, 30]. Low levels of gga-miR-26a and gga-miR-181a have been associated with suppression of MDV-induced tumors [31]. MDV-miR-M4 is known to complement miR-155 in initiating MD lymphomas, although the underlying mechanisms, especially the role of host miRNAs, have not completely elucidated. The exact biological relevance of gga-miRNA-155 in MD tumorigenesis needs to be confirmed. To this end, we overexpressed and inhibited gga-miR-155 in an MDV-transformed cell line using mimics and inhibitors respectively, and analyzed their growth, proliferation, apoptosis and invasiveness to explore the possible role of gga-miR-155 in MDV-mediated tumorigenesis. Furthermore, we predicted and demonstrated that Retinoid Acid Receptor-Related Orphan Receptor Alpha (RORA) is one of the targets of gga-miR-155, and the gga-miR-155 regulated the proliferation, cell cycle, apoptosis and invasiveness of MSB1 cells by targeting RORA.

## Results

### Gga-miR-155 promotes the proliferation and cell cycle progression of MSB1 cells

**Fig. 1:**
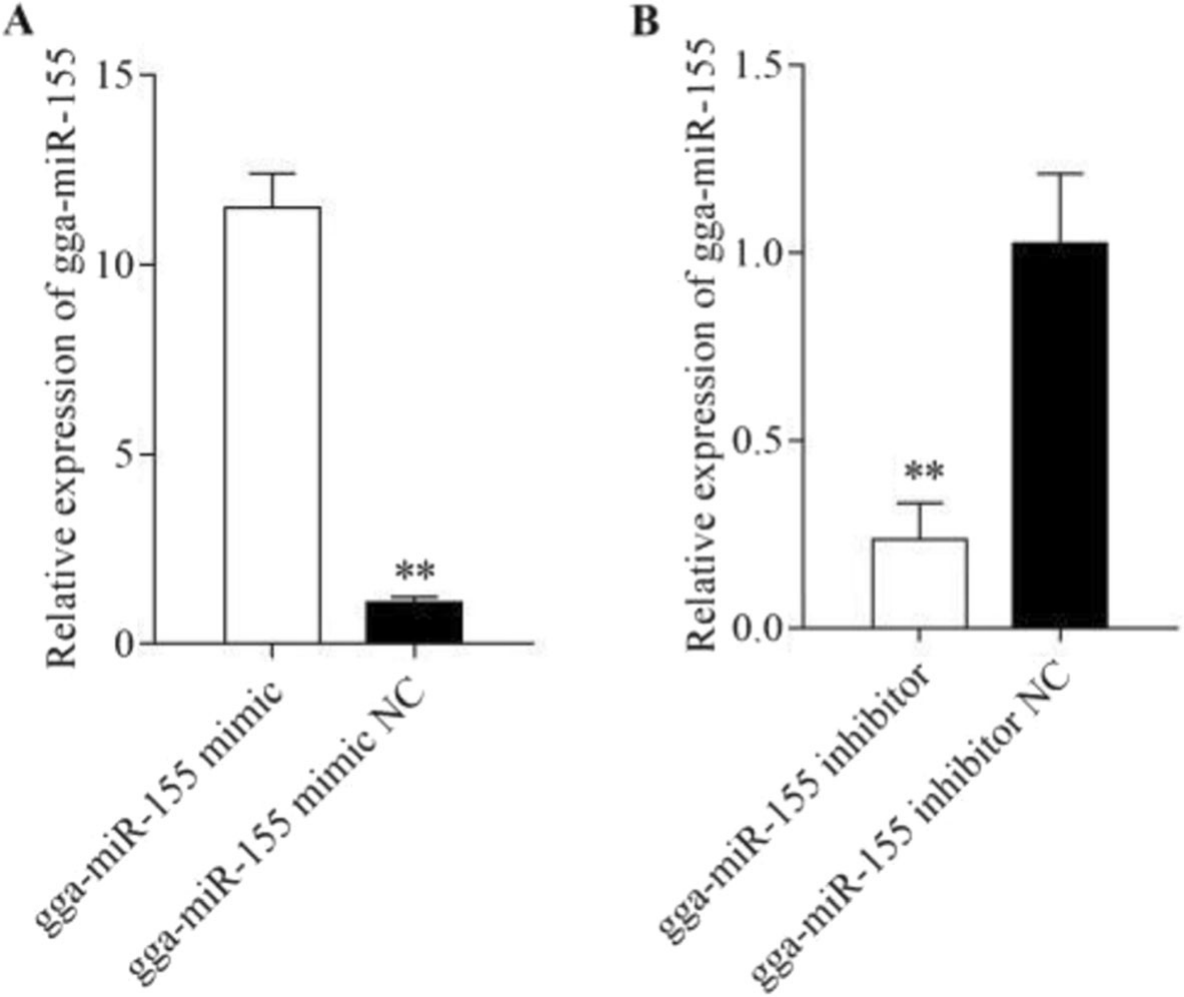
The expression levels of gga-miR-155 in MSB1 cells transfected with (**a**) gga-miR-155 mimic and (**b**) gga-miR-155 inhibitor. ** *P* <0.01, * *P* <0.05

**Fig. 2:**
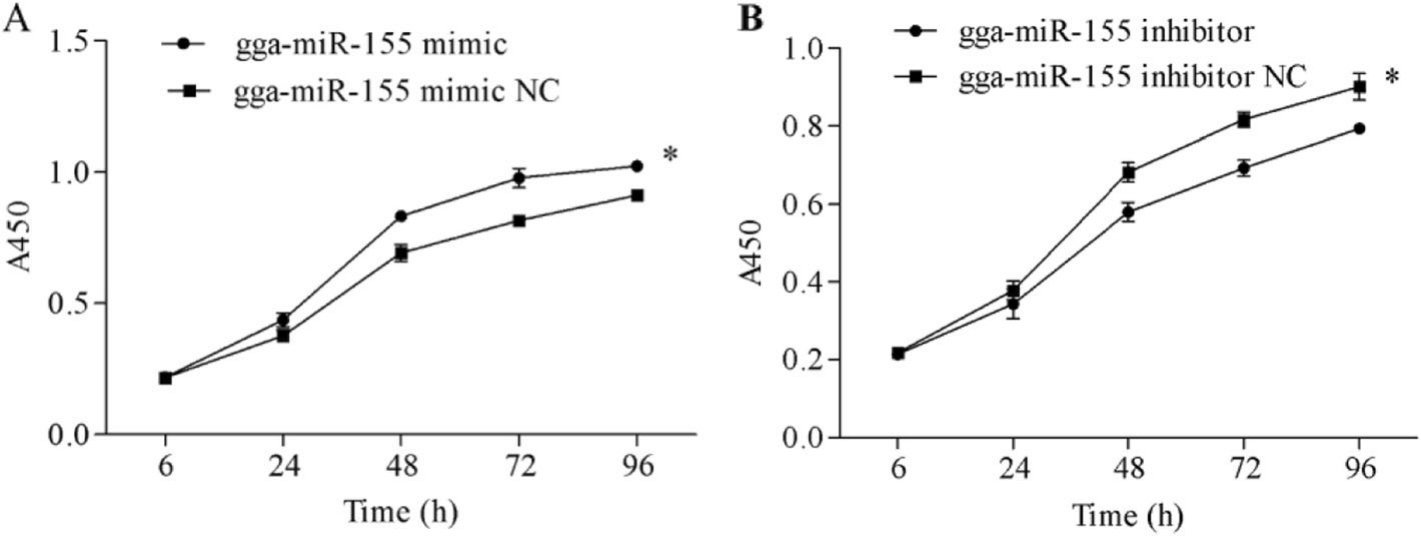
gga-miR-155 promoted proliferation of MSB1 cells Time-dependent growth curve of MSB1 cells transfected with (**a**) gga-miR-155 mimic and (**b**) gga-miR-155 inhibitor and their respective controls. * *P* <0.05 versus control

**Fig. 3:**
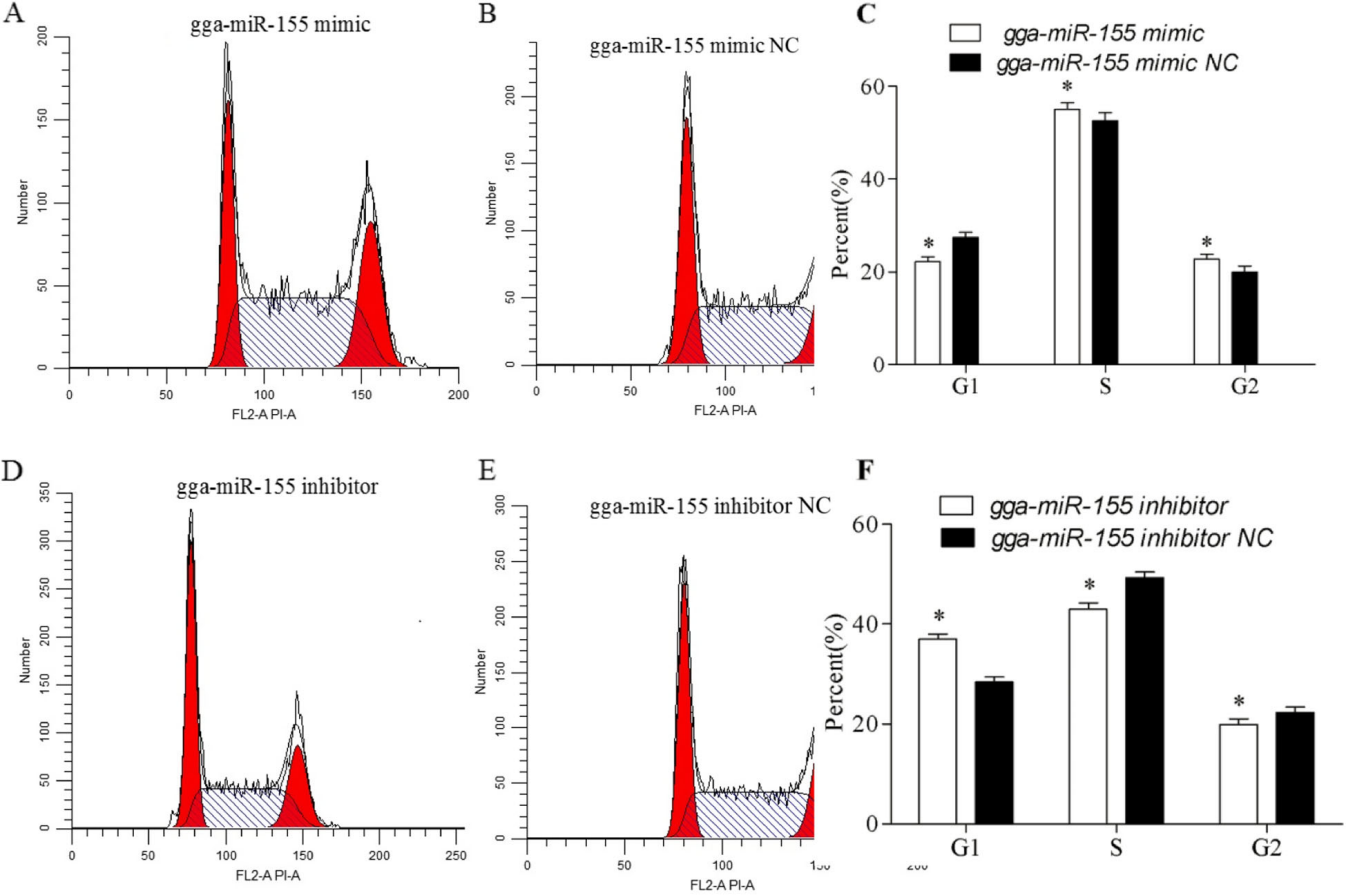
gga-miR-155 accelerated progression through the cell cycle. Flow cytometry histograms show the proportion of cells in the different phases of the cell cycle following transfection with (**a**) gga-miR-155 mimic, (**b**) gga-miR-155 mimic NC, (**d**) gga-miR-155 inhibitor and (**e**) gga-miR-155 inhibitor NC. Bar graphs comparing the percentage of cells in the G1, S and G2 phases of the (**c**) gga-miR-155 mimic/NC and (**d**) gga-miR-155 inhibitor/NC transfected groups.* *P* <0.05

To determine the role of gga-miR-155 in MDV-transformed T cells, we respectively overexpressed and downregulated the miRNA in MSB1 cells using mimics and inhibitors (Fig. 1). While high levels of gga-miR-155 enhanced the proliferative capacity of the MSB1 cells compared to the controls (*P* < 0.05), its downregulation had the opposite effects (P < 0.05) (Fig. 2). Furthermore, analysis of the cell cycle distribution showed that the proportion of cells in the G1 phase decreased significantly in the gga-miR-155 mimics group compared to the respective control, and that in the S and G2 phases increased. Upon gga-miR-155 inhibition however, the cells accumulated in the G1 phase, with a concomitant decrease in the proportion of cells in the S and G2 phases (*p* < 0.05) (Fig. 3). Consistent with these results, gga-miR-155 accelerated MSB1 cell proliferation and cell cycle progression.

### Gga-miR-155 inhibits apoptosis of MSB1 cells

**Fig. 4:**
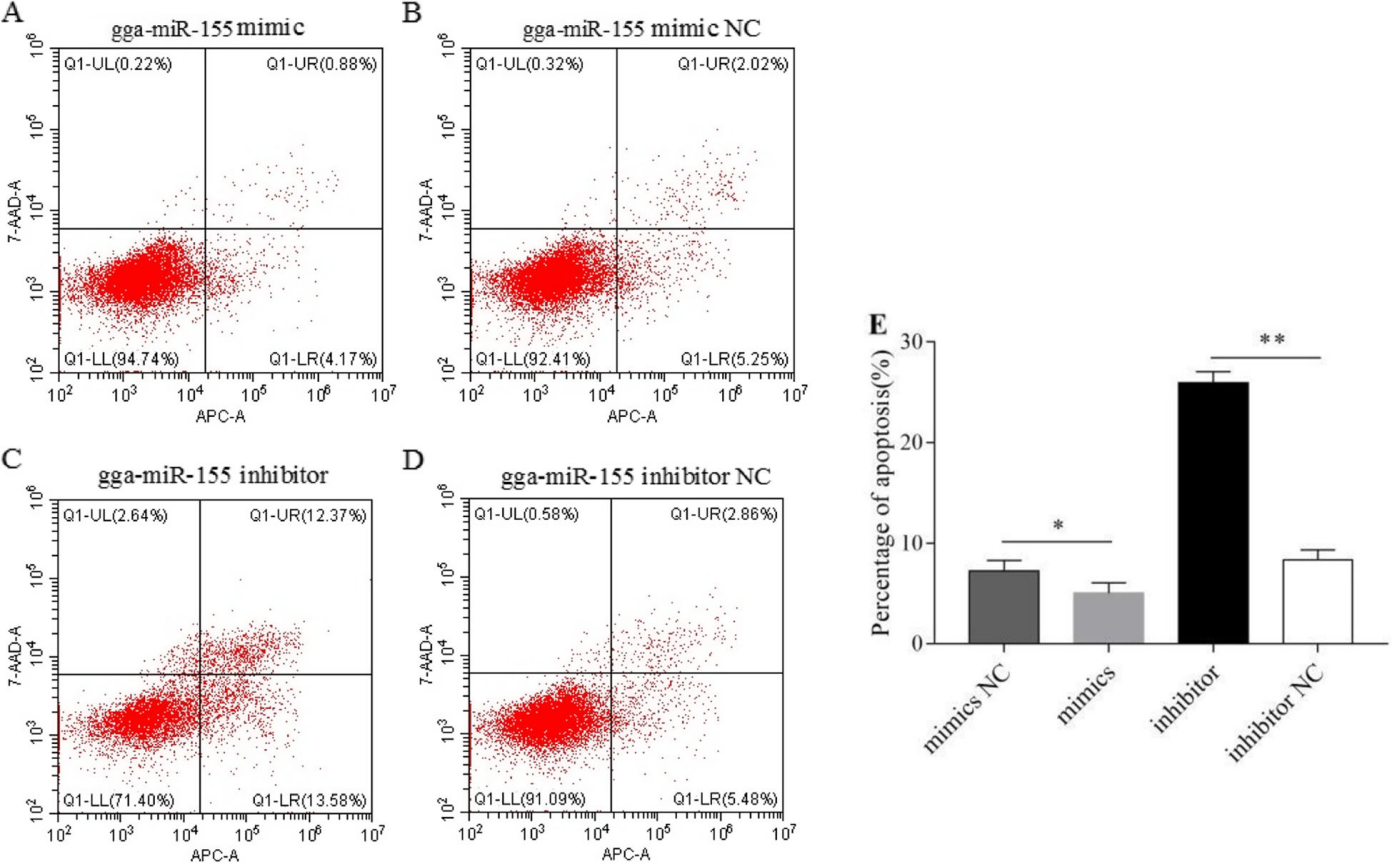
gga-miR-155 blocked apoptosis in MSB1 cells. Flow cytometry dot plots showing the percentage of live and apoptotic cells in the (**a**) gga-miR-155 mimic, (**b**) gga-miR-155 mimic NC, (**c**) gga-miR-155 inhibitor and (**d**) gga-miR-155 inhibitor NC groups. (**e**) Percentage of apoptotic cells in the different groups. * *P* <0.05

To determine the effect of gga-miR-155 on apoptosis, the percentage of apoptotic MSB1 cells was evaluated 48 h after transfecting with the different constructs. The proportion of apoptotic cells was significantly lower among those transfected with gga-miR-155 mimics compared to the control. Furthermore, the gga-miR-155 inhibitor significantly increased the proportion of apoptotic cells compared to the inhibitor NC (*P* < 0.05, Fig. 4). These results indicated that gga-miR-155 can inhibit apoptosis of MSB1 cells.

### Gga-miR-155 promotes migration and invasion of MSB1 cells

**Fig. 5:**
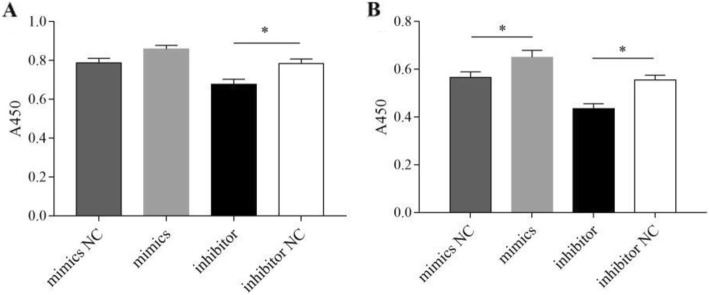
Effects of gga-miR-155 on the migrate and invasion of MSB1 cells. Bar graphs showing the migration and invasion rates of MSB1cells transfected with (**a**) The migration of MSB1 cell treated with gga-miR-155 mimic/NC &inhibitor/NC (**b**) The invasion of MSB1 cell treated with gga-miR-155 mimic/NC &inhibitor/NC. * *P* <0.05

The migration and invasiveness of MSB1 cells were also assessed following transfection with the different constructs. As shown in Fig. 5, overexpression of gga-miR-155 slightly increased the migration capacity of the MSB1 cells (*P* > 0.05), while the gga-miR-155 inhibitor significantly decreased the proportion of migrating cells (*P* < 0.05, Fig. 5a). Furthermore, the invasive capacity of MSB1 cells transfected with gga-miR-155 mimics was notably increased (*P* < 0.05), and that of cells transfected with gga-miR-155 inhibitors was significantly decreased (P < 0.05, Fig. 5b). Thus, gga-miR-155 also promotes the migration and invasion of MDV-transformed cells.

### Gga-miR-155 suppresses RORA expression by binding to its 3′ UTR sequence

**Fig. 6:**
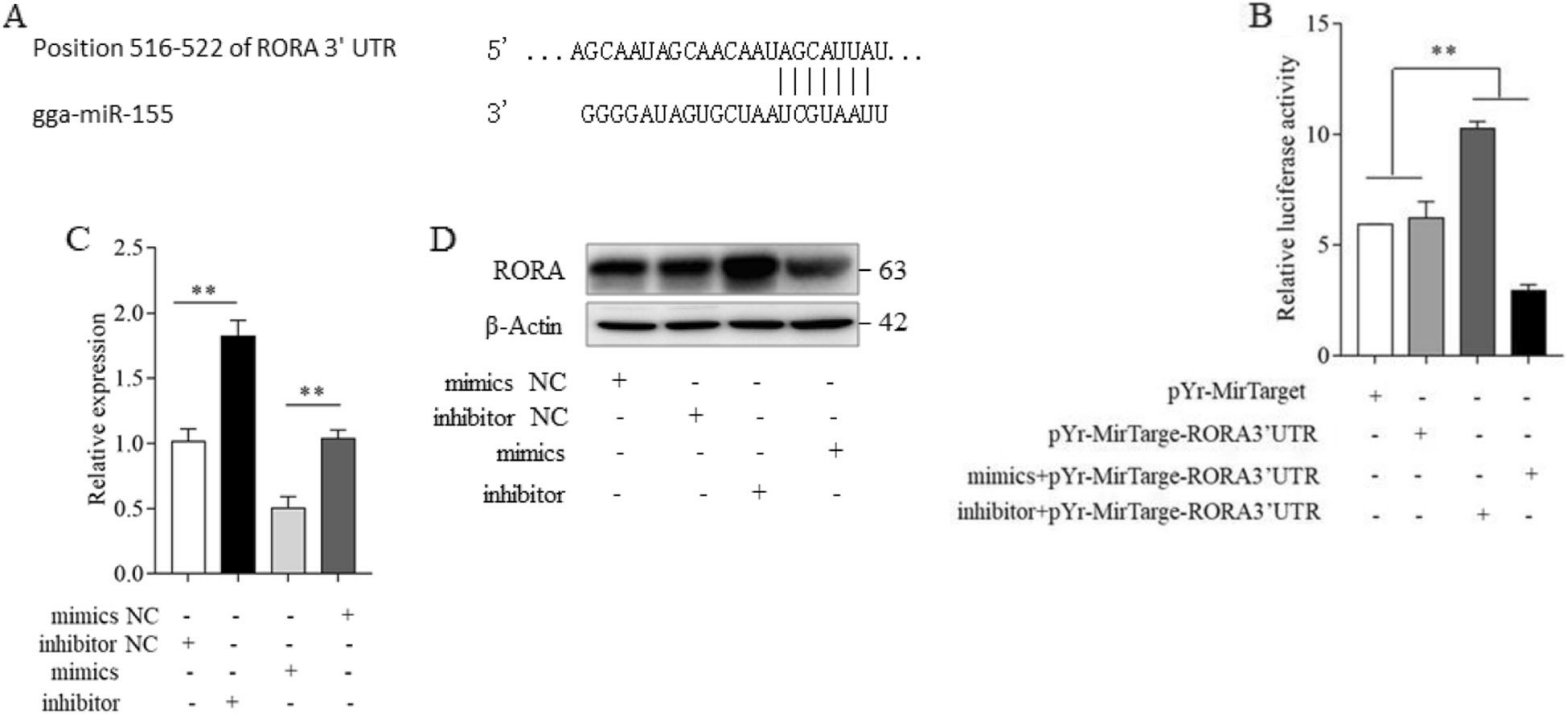
gga-miR-155 directly binds to and regulates the expression of RORA gene in MSB1 cells. **a** The potential gga-miR-155 seed region at the binding site in RORA 3’ UTR (position 516-522) was predicted by TargetScan. **b** Relative luciferase activity in the different groups. **c** Expression levels of RORA mRNA in the different groups. **d** Immunoblot showing expression of RORA protein in the different groups. Error bars indicate the standard deviation from three independent replicates. ** *P* <0.01, * *P* <0.05

Previous studies have identified the tumor suppressor RORA as a putative target of miR-155 [32]. To validate this surmise, we screened for the putative target genes of miR-155 using TargetScan (release 6.2, http://www.targetscan.org/) (Fig. 6a). The direct binding of gga-miR-155 to the 3′-UTR of the chicken RORA gene was assessed by the dual luciferase reporter assay (DLRA). Briefly, HEK293T cells were transfected with pYr-MirTarget-RORA 3′-UTR with or without the gga-miR-155 mimics or gga-miR-155 inhibitors. As shown in Fig. 6b, the relative luciferase activity of the reporter significantly decreased in the presence of gga-miR-155 mimics and increased when co-transfected with gga-miR-155 inhibitor. we next determined whether altering the expression levels of gga-miR-155 affected that of RORA in the MSB1 cells. In agreement with our hypothesis, RORA mRNA (Fig. 6c) and protein (Fig. 6d) levels respectively decreased and increased in the cells transfected with gga-miR-155 mimic and gga-miR-155 inhibitor. Therefore, gga-miR-155 suppresses RORA both transcriptionally and post-transcriptionally in the MSB1 cells. Taken together, the RORA gene is a putative target gene of gga-miR-155, which binds to the former’s 3′-UTR region.

## Discussion

MicroRNAs are conservative, single-stranded non-coding small molecular RNA ~ 22–25 nucleotides long, with a characteristic hairpin structure that is synthesized by the RNA endonucleases Drosha and Dicer. The 5′-terminal seed sequences of mature miRNAs regulate target gene expression at the post-transcriptional level by binding to the 3′-UTR of the target mRNAs, which results in their degradation or translational suppression [29, 33]. The biological role of miRNAs has gained considerable attention in recent years, and several have been identified as oncogenes or tumor suppressor genes that regulate proliferation, differentiation, apoptosis and migration of cancer cells [34, 35]. Therefore, miRNAs are potential markers for the diagnosis, prognosis, classification, staging and therapeutic monitoring of cancers. MiRNA-155 for instance is associated with the occurrence and development of renal cancer [36], glioma [37], lung cancer [38], colon cancer [39, 40] and other malignancies [41–44].

To elucidate the role of miR-155 in the MDV-transformed cells, we manipulated its expression levels using specific mimics and inhibitors. Overexpression of gga-miR-155 significantly increased the proliferation of MSB1 cells, accelerated progression through the cell cycle, decreased apoptosis, and promoted their migration and invasiveness in vitro. Not surprisingly, down-regulating gga-miR-155 had the opposite effects. These findings are interesting considering the fact that although gga-miR-155 was down-regulated in MDV transformed T lymphocyte lines, spleen tumor tissues and liver lymphoma after MDV infection, it could promote proliferation, migration and invasiveness of MSB1 cells and inhibit apoptosis.

Retinoid acid receptor-related orphan receptor alpha (RORA) is a member of the nuclear receptor protein superfamily that regulates circadian rhythm regulator, metabolism, immune responses and inflammatory diseases [45, 46]. It is also a tumor suppressor and therefore inactivated during malignant transformation, and tumor initiation and metastasis [47, 48]. Several targets of miRNA-155 have been identified in recent years, such as the BRG1 [49] and FOXO3a [50] in lymphoma, and SOCS1 in severe acute pancreatitis [51]. Parnas et al. predicted 9 common target genes of MDV1-miR-M4, KSHV-miR-K11 and HAS-miR-155, and 4 of MDV1-miR-M4 and HAS-miR-155 in human B cells and chicken T cells, which includes RORA [32]. In the present study also, we identified RORA as one of the putative targets of gga-miRNA-155 through bioinformatics analysis. Furthermore, luciferase reporter assay revealed that gga-miR-155 could directly target the 3′-UTR of RORA. Taking together all these findings, we can conclude that RORA is a direct target of gga-miR-155 in MDV-induced tumorigenesis.

## Conclusions

The gga-miRNA-155 promotes proliferation, migration and invasiveness of MSB1 cells and inhibits their apoptosis via RORA by targeting the latter’s 3′-UTR region. Since this contradicts the role of other miRNAs that are downregulated during MD tumorigenesis, it remains to be elucidated whether the low expression level of gga-miRNA-155 in MD tumor cells or tissues is correlated to other non-coding RNAs.

## Methods

### Cell culture and transfection

The MDV-transformed chicken lymphoblastoid cell line MDCC-MSB1 was purchased from Shanghai Kindu Biotechnology Co. Ltd. The cells were cultured at 37 °C under 5% CO_2_ in RPMI 1640 medium (Gibco, USA) supplemented with 10% fetal calf serum (Gibco, USA), 10% tryptose phosphate broth (Sigma, USA) and 1% penicillin-streptomycin solution (HyClone, USA). The gga-miR-155 mimic, gga-miR-155 inhibitor and their respective negative controls were synthesized by RiboBio Corporation (China). The constructs were transfected using FuGENE® HD (Promega, USA) according to the manufacturer’s instructions. Briefly, the cells were seeded in 6-well plates at the density of 3 × 10^5^ per well, and transfected with 50 nM gga-miR-155 mimic or 200 nM gga-miR-155 inhibitor and their respective controls (NC). The transfection efficiency was validated after 48 h by RT-qPCR.

### Stem-loop quantitative real- time PCR (qRT- PCR)

Total RNA was extracted from the cultured cells using TRIzol reagent (Invitrogen, USA) according to the manufacturer’s protocol, and quantified using the NanoDrop ND-2000 Spectrophotometer (Thermo, USA). Reverse transcription was performed using the miRNA-specific stem-loop reverse-transcription primer (Sangon, Shanghai, China) using 1 μg total RNA. Real time qPCR was performed using miScript SYBR Green PCR kit (Qiagen,USA) in the ABI 7900 PCR Detection System (Applied Biosysterm, USA). The cycling parameters were as follows: 50 °C for 2 min, 95 °C for 10 min, and 40 cycles of 95 °C for 30 s and 60 °C for 1 min. The relative target gene expression (2^−ΔΔCt^) was normalized to that of U6 endogenous small nuclear RNA. The primer sequences are shown in Table 1.

**Table 1:** Sequences of primers used for qRT-PCR assays

Name	Primer Sequences (5′ → 3′)
U6 RT prime	GTCGTATCCAGTGCAGGGTCCGAGGTATTCGCACTGGATACGACCGATACA
U6 Forward primer	CGCTTCGGCAGCACATATAC −3′
gga-miR-155 RT prime	GTCGTATCCAGTGCAGGGTCCGAGGTATTCGCACTGGATACGACCCCCTATC
gga-miR-155 Forward primer	TGCGCTTAATGCTAATCGTGAT
gga-miR-155/U6 Reverse primer	CCAGTGCAGGGTCCGAGGTATT
gga -β-actin Forward primer	AGAAGGAGATCACAGCCCT
ggaβ-actin Reverse primer	GGGTCCGGATTCATCGTACT
RORA Forward primer	GACCTCTCCAACTGTGTCCA
RORA Reverse primer	GCCACATTACCTCCCTTTGC

### Target genes prediction and luciferase reporter assay

The target genes of gga-miR-155 were predicted using the online tools TargetScan (http://www.targetscan.org) and miRDB (http://mirdb.org/miRDB/). RORA 3′-UTR sequences containing the putative gga-miR-155 binding sites were amplified and cloned into the pYr-MirTarget luciferase reporter vector. The primers used are listed in Table 2. HEK293T cells in the logarithmic growth phase were seeded into 12-well plates and cultured overnight, and co-transfected with the RORA 3′-UTR reporter vector and gga-miR-155 mimics or gga-miR-155 inhibitor using Lipofectamine™ 2000. Luciferase activity was measured 48 h after co-transfection using the Double Luciferase Reporter Gene Assay kit (Promega) according to the manufacturer’s instructions. The ratio of renilla and firefly luciferase intensities was calculated. The assay was performed thrice.

**Table 2:** Sequences of primers used for amplification of RORA 3′-UTR

Name	Primer Sequences (5′ → 3′)
RORA3′-UTR Forward primer	TAGGCGATCGCTCGAGCATTGTTTCATGAAGGACGAT
RORA 3’-UTR Reverse primer	TAGGCGATCGCTCGAGCATTGTTTCATGAAGGACGAT

### Western blotting

Total proteins were extracted from MSB1 cells 48 h post-transfection using radio immunoprecipitation assay (RIPA) lysis buffer supplemented with protease and phosphatase inhibitors. The concentration of proteins was determined by the BCA assay (BCA Protein Assay Kit, Beyotime, Shanghai, China), and 20 μg protein per sample was denatured in loading buffer by boiling for 3~5 min, and separated by 10% SDS-PAGE. The resulting bands were electro-transferred to polyvinylidene difluoride (PVDF) membrane at 100 mA over 1.5 h. After blocking with 4% BSA for 1 h, the membranes were incubated overnight with primary antibodies against RORA (1:1000, Abcam, ab60134) and β-actin (1:1000, Abcam, ab8226), followed by HRP-conjugated anti-rabbit IgG (1:1000) and anti-mouse IgG (1:1000) (Bayotime) respectively. The positive proteins bands were detected using a chemiluminescence system (Bio-Rad Clarity Western ECL; Bio-Rad Laboratories Inc.), and the grayscale values were quantified using ImageJ. The density of the RORA bands was standardized to that of β-actin.

### Cell proliferation assay

Suitably transfected MSB1 cells were harvested after 6 h, and seeded into 96-well plates at the density of 4000 cells/well. Twenty microliters of the Cell Counting Kit 8 reagent (CCK 8, Biosharp Biotech, China) was added to each well after 6, 24, 48, 72 and 96 h of culture, and the optical density (OD) was measured at 450 nm using an ELISA reader (Thermo, USA) following a 4 h incubation.

### Cell cycle assay

The cell cycle profile was analyzed using the Cell Cycle Detection Kit (KeyGen, China) according to the manufacturer’s instructions. Briefly, the cells harvested 48 h after transfection were washed twice with cold PBS, and re-suspended in 100 μl PBS. After fixing with 70% ice-cold ethanol for 4 h at 4 °C, the cells were rinsed twice with cold PBS, incubated with 100 μl RNase (50 μg/mL; Sigma, USA) for 30 min at 37 °C, and finally stained with 400 μl PI (50 μg/ml) in the dark at 4 °C for 30 min. The stained cells were analyzed by flow cytometry (BD bioscience, USA). Each sample was tested in triplicates.

### Cell migration and invasion assay

The in vitro migration and invasion of MSB1 cells were analyzed 48 h after transfection using the transwell method. For the migration assay, RPMI 1640 medium containing 10% FBS was dispensed into the lower chambers of transwell inserts (8 μm pore size; Corning 3422, USA) placed in a 24-well plate. After 1 h, 200 μL of the cell suspension (6 × 10^5^ cell/ml) in serum free RMPI 1640 was seeded in the upper chambers of each insert. After incubating the cells for 24 h at 37 °C under 5% CO_2_, the transwells were removed, and 60 μL CCK-8 reagent (Biosharp Biotech, China) was added to each well. The viability of the migrated cells was assessed as already described. The invasiveness of MSB1 was assayed as above, except that the seeding density of the cells was 1 × 10^5^/well, and the upper chambers were pre-coated with Matrigel (BD Bioscience, USA). Each sample was tested in triplicates.

### Annexin/7-AAD staining

Apoptosis in the MSB1 cells was evaluated by AnnexinV-APC/7-AAD staining and flow cytometry (CytoFLEX; Beckman Coulter Inc., USA). Transfected cells were harvested after 48 h, washed twice with cold PBS, and stained using the AnnexinV-APC/7-AAD Cell Apoptosis Detection kit (NanJing KeyGen Biotech Co.,Ltd., China) according to the manufacturer’s instructions. The cells were resuspended in 500 μl binding buffer,and incubated with 5 μL each of AnnexinV-APC and 7-AAD for 15 min in the dark at room temperature. The stained samples were analyzed by flow cytometry, and the percentage of apoptotic cells was calculated.

### Statistical analysis

SPSS 19.0 and GraphPad Prism (Version 6.0) were used for data analysis. Data were expressed as mean ± SD. Two groups were compared using Student’s t-test, and multiple groups with the one-way ANOVA and LSD tests. *P* values < 0.05 were considered statistically significant.

## Data Availability

The datasets used and/or analyzed during the current study are available from the corresponding author on reasonable request.

## Abbreviations

DMEM: Dulbecco’s modified Eagle’s medium
DMSO: Dimethyl sulfoxide
FBS: Fetal bovine serum
MD: Marek’s disease
MDV: Marek’s disease virus
miRNAs: Micrornas
qRT-PCR: Quantitative Real-time Polymerase Chain Reaction
SDS-PAGE: Sodium dodecyl sulfate polyacrylamide gel electrophoresis
TPB: Tryptose phosphate broth
UTR: Untranslated region.; RORA: Retinoid acid-related orphan receptor A
